# A Dual-Path CNN and Transformer Network for Continuous Pavement Crack Detection

**DOI:** 10.3390/s26113286

**Published:** 2026-05-22

**Authors:** Jinhe Zhang, Shangyu Sun, Weidong Song, Yuxuan Li, Qiaoshuang Teng

**Affiliations:** 1 School of Geomatics, Liaoning Technical University, Fuxin 123000, Chinasouleleven@163.com (Q.T.); 2 Collaborative Innovation Institute of Geospatial Information Service, Liaoning Technical University, Fuxin 123000, China; 3 State Key Laboratory of Information Engineering in Surveying, Mapping and Remote Sensing, Wuhan University, Wuhan 430079, China

**Keywords:** crack segmentation, deep learning, convolutional neural network, Vision Transformer

## Abstract

Cracks are among the most common pavement distresses, and their timely detection is crucial for road maintenance. Existing methods struggle to completely capture elongated and irregular cracks, often resulting in fragmented detection outputs, which leads to the inaccurate assessment of crack length and affects the reliability of pavement condition evaluation. To address this issue, this paper proposes a dual-path crack segmentation network that integrates CNN and Transformers. The CNN branch incorporates a dynamic multi-branch convolution module to enhance the directional perception and structural modeling of elongated cracks. The Transformer branch employs a lightweight DCNv4 module to replace traditional self-attention mechanisms, effectively capturing long-range dependencies while reducing computational complexity. A multi-path fusion module is designed to achieve the collaborative enhancement of dual-path features, improving the semantic representation of continuous crack regions. Additionally, a combined loss function of BCE and Dice is adopted to alleviate the severe class imbalance between crack and background pixels, further improving the completeness of crack segmentation. Experiments on four datasets, including CFD, DeepCrack537, Gaps384, and Crack500, demonstrate that the proposed model outperforms all compared methods in terms of F-score and mIoU. Ablation studies further validate the effectiveness of the dual-path architecture and its key modules in improving performance. Furthermore, in field validation on real road scenarios, the pavement condition index (PCI) calculated based on the proposed method shows an average deviation of only 0.81 compared to manually interpreted ground truth, demonstrating the practical value of continuous crack detection for pavement maintenance assessment.

## 1. Introduction

With the development of the transportation industry, roads have become critical infrastructure directly affecting traffic safety and efficiency. However, during long-term operation, roads often experience distress due to factors such as overloading, temperature changes, rainwater erosion, and material aging, with cracks being the most common [1]. If cracks are not detected and repaired in time, they can further expand into severe damage such as potholes and fractures, affecting pavement strength and lifespan [2], and even endangering driving safety [3]. Therefore, efficient and accurate crack detection is crucial for road maintenance and safety assurance.

Currently, most regions still rely on manual inspection, which suffers from strong subjectivity, low efficiency, and high costs, making it difficult to meet large-scale road inspection demands. To address the limitations of manual inspection, researchers proposed a series of traditional image-processing methods, including edge detection [4], threshold segmentation [5], filtering and feature enhancement [6], morphological operations [7], region growing [8], and projection fitting [9]. These methods achieved promising results in specific scenarios, but their performance remains unstable under uneven illumination, complex backgrounds, or severe noise interference, making them insufficient for practical large-scale applications.

With the rapid development of deep learning technology, particularly convolutional neural networks in image recognition [10,11,12], an increasing number of studies have begun applying the technology to pavement crack detection tasks [13,14,15]. These methods can achieve accurate crack identification through the automatic learning of crack feature patterns in images and possess strong robustness and generalization capability. However, for crack images, these annotations are inherently subjective and noisy due to complex crack patterns, ambiguous boundaries, and annotator inconsistency. Zhang et al. [16] proposed an uncertainty-based selective training and prediction framework called SelectSeg, combining deep ensembles, uncertainty-guided filtering, and semi-supervised learning to address noisy crack annotations, demonstrating the importance of annotation quality for segmentation reliability. Zim et al. [17] proposed a lightweight crack segmentation network called EfficientCrackNet, combining edge extraction modules, subspace attention modules, and MobileViT to effectively enhance edge perception and global feature modeling capability. Chen et al. [18] proposed COPNet for pavement crack segmentation, achieving the simultaneous detection of cracks at different scales and more precise segmentation through scale-aware channel attention and cross-scale patch attention mechanisms. Sun et al. [19] proposed DUCTNet for road crack segmentation in complex scenes, effectively improving crack-background discrimination and fine crack detection capability through deep nested structures, competitive fusion modules, and high-density attention mechanisms. Qu et al. [20] proposed DCFENet for pavement crack detection, effectively improving crack topology modeling capability and edge detail segmentation accuracy through multi-directional enhanced convolution, multi-scale fusion attention mechanisms, and directional connectivity loss. Wang et al. [21] proposed LPCD-MSMD for pavement crack detection, maintaining high detection accuracy while significantly compressing model parameters through cascaded U-Net structure, coordinate attention mechanism, and multi-scale semantic map distillation strategy, suitable for crack detection tasks in resource-constrained scenarios. Chen et al. [22] proposed a crack recognition network called HACNet, which uses dilated convolutions with different dilation rates to expand the receptive field and aggregate multi-scale features through hybrid connections, achieving accurate crack segmentation with fewer parameters. CNNs are constrained by their fixed receptive fields and local connectivity, making it difficult for convolutional kernels to adapt to the linear structures of elongated cracks. Additionally, the model cannot establish long-range dependencies between distant parts of cracks, often producing discontinuous segmentation results with frequent breakpoints along long cracks.

In recent years, Transformer architecture has gradually become an important research direction in computer vision due to its excellent global modeling capability and self-attention mechanism. Compared to CNN, Transformer can model long-distance dependencies and more effectively capture global structural information in images, performing excellently in tasks such as image classification, object detection, and semantic segmentation. It has also been introduced to crack detection to enhance the model’s perception capability for complex backgrounds, and multi-scale structures. Liu et al. [23] proposed a Transformer-based crack segmentation network called CrackFormer, introducing self-attention modules and scaled attention modules in the SegNet architecture to enhance inter-channel context modeling and cross-layer feature alignment capability, improving fine-grained crack detection effects. Xiao et al. [24] proposed HWFormer based on a hybrid window attention mechanism, which combines local window and global window attention modules to effectively capture multi-scale context information and improve crack edge representation capability. Sun et al. [25] proposed MorFormer, a morphology-aware network that effectively enhances the modeling of different crack morphological features and the fusion of multi-scale context information through morphology-aware modules and cascaded fusion modules. Xiong et al. [26] proposed FCTNet, a dual-encoding crack segmentation network combining atrous spatial pyramid pooling and Transformer, introducing long–short distance attention mechanisms and attention weight cross-fusion modules to achieve the efficient fusion of local and global features, improving segmentation performance in complex scenes. Hu et al. [27] proposed CCDFormer based on cross-scale context decoupling and reconstruction mechanisms, introducing decoupled context encoding modules to extract local and global features, and enhancing feature representation and detail restoration capability through cross-scale context reconstruction modules. Compared to traditional convolutional neural networks, Transformer performs better in handling long-range dependencies, but it lacks local relationship capture and has high computational complexity.

In summary, pavement cracks typically exhibit elongated and irregular morphological characteristics, where local and global features are equally crucial for accurate segmentation. CNN-based methods excel at extracting local detail features and are suitable for crack boundary localization, but are limited by their restricted receptive fields, making it difficult to establish spatial correlations among distant crack regions and thus prone to discontinuities in the detection of long cracks. Transformer-based methods can capture long-range dependencies and global contextual information, which is favorable for continuous detection, yet they lack the fine-grained perception of local geometric structures and perform inadequately on tiny cracks and edge details. Recognizing this complementarity, several recent studies have explored dual-path architectures that combine CNN and Transformer branches for crack segmentation. However, these existing dual-path networks share three common limitations. First, their CNN branches typically employ standard isotropic convolutions, which lack directional adaptability for the anisotropic geometry of elongated cracks. Second, their Transformer branches generally rely on standard multi-head self-attention or fixed-window attention, which uniformly compute attention over all spatial positions including irrelevant background regions, resulting in both computational waste and difficulty in connecting fragmented crack segments. Third, their feature fusion strategies are mostly limited to simple concatenation or element-wise addition, which do not fully exploit the complementarity of the two branches.

To address these limitations, this paper proposes a dual-path parallel network architecture that empirically improves the continuity and completeness of crack segmentation through stage-wise feature fusion with specifically designed modules. The main contributions are summarized as follows:

(1) A dual-path CNN–Transformer parallel network architecture is proposed. Unlike existing dual-path methods that rely on generic modules, the proposed CNN branch incorporates a dynamic multi-branch convolution module (DMBConv) employing multi-directional learnable convolutional kernels to enhance the directional adaptive perception of elongated cracks. In the Transformer branch, DCNv4 is adopted to replace the traditional self-attention mechanism, enabling adaptive focus on critical crack regions through deformable sparse sampling while reducing computational complexity.

(2) A feature fusion block is designed with three parallel pathways including local attention, global attention, and a feature selection branch with learnable task embedding and a channel selection matrix, achieving deep interaction and the adaptive integration of dual-path features. This goes beyond the simple concatenation or addition used in prior dual-path networks.

(3) A combined loss function of BCE and Dice is adopted to introduce regional continuity constraints on top of pixel-level classification, and a set of continuity evaluation metrics (BD, ASL, LPR, FI, CI) are employed to quantitatively assess crack detection completeness, bridging the gap between segmentation accuracy and engineering-level pavement condition assessment.

(4) Extensive experiments are conducted on four public crack datasets: CFD, DeepCrack537, Gaps384, and Crack500. The proposed method achieves the best performance among all compared methods in both F-score and mIoU metrics and generates more continuous and complete crack segmentation results. Field validation on real road sections further demonstrates the link between improved continuity and more accurate PCI calculation.

## 2. Related Work

### 2.1. CNN-Based Semantic Segmentation Methods

Convolutional neural networks have always been one of the core technologies in image semantic segmentation. Since FCN [28] was first proposed, CNN-based methods have been widely applied to various segmentation tasks. Early methods such as UNet [29] and SegNet [30] adopted classic encoder–decoder structures and effectively alleviated information loss during upsampling through skip connections, laying the foundation for subsequent model designs. However, models based on FCN have limited receptive fields and find it difficult to learn remote dependency relationships. To address this issue, the DeepLab [31] series introduced atrous convolution and atrous spatial pyramid pooling structures, which expand the receptive field without losing resolution, significantly enhancing contextual awareness. PSPNet [32] utilized pyramid pooling strategies to obtain global context information at different scales, further enhancing the model’s understanding capability for complex scenes.

Meanwhile, attention mechanisms have been introduced into CNN structures to improve feature selection accuracy and the ability to focus on key regions. BAM [33] embedded channel and spatial attention in parallel into residual structures, enhancing key features in information flow with lightweight computation; CBAM [34] adopted sequential attention based on this, guiding the network to focus more on target regions. SKNet [35] introduced dynamic kernel selection mechanisms, demonstrating the potential of attention mechanisms in multi-scale modeling. Following a similar idea of adapting kernel behavior to target geometry, Qi et al. [36] proposed dynamic snake convolution for tubular structure segmentation, in which the convolutional kernel iteratively deforms along the centerline trajectory of the target structure, providing strong continuity-aware feature extraction for blood vessels and roads, this idea is also relevant to elongated crack detection. In addition, regarding the requirement for boundary accuracy in segmentation tasks, BASNet [37] strengthened the model’s perception of target contours through edge supervision branches and improved segmentation accuracy with residual refinement strategies. EGNet [38] introduced multi-layer edge guidance modules, fusing shallow boundary information into deep semantic representations to achieve edge-semantic joint enhancement. PIDNet [39] constructed a three-branch structure of parsing, integration, and detail, where the dedicated boundary branch with supervision mechanisms significantly improved segmentation quality in edge regions while ensuring semantic consistency.

In the field of crack segmentation, Choi et al. [40] designed SDDNet, which uses depthwise separable convolutions to significantly reduce computational costs, enabling real-time crack segmentation. Jiang et al. [41] proposed HDCBNet, leveraging mixed dilated convolutional blocks to enhance multi-scale feature extraction. Chu et al. [42] introduced TinyCrackNet, designing a multi-scale feature fusion network combined with attention mechanisms to effectively improve the segmentation accuracy of fine cracks. Sun et al. [43] proposed DMANet, which integrates a multi-scale attention module based on DeepLabv3+, achieving more refined crack segmentation by dynamically allocating weights to features from different layers.

Despite the excellent performance of CNN-based methods in feature extraction and training stability, their inherent local perception mechanism limits their ability to model long-range dependencies. Existing methods mainly rely on deepening the network or stacking modules to expand the receptive field, which not only leads to parameter redundancy but can also result in the loss of local details.

### 2.2. Transformer-Based Semantic Segmentation Methods

With the proposal of Vision Transformer, Transformer architecture has gradually become an important research direction in semantic segmentation. Its core advantage lies in global modeling capability, being able to capture long-range dependencies, thereby compensating for the limitations of convolutional neural networks in receptive field and structural modeling.

ViT [44], the earliest Transformer model applied to image tasks, divides images into fixed-size patches and inputs them into self-attention mechanisms for modeling. Although it achieved remarkable performance, its effectiveness in semantic segmentation was limited due to the lack of multi-scale features and spatial detail modeling capability. Therefore, SETR [45] first introduced Transformer to segmentation tasks, using a pure Transformer encoder to replace the CNN backbone and restore spatial structure through multi-layer decoding modules, validating the feasibility of this structure in dense prediction tasks. Subsequently, numerous improved methods were proposed. Swin Transformer [46] achieved efficient feature modeling and resolution recovery by constructing hierarchical window self-attention mechanisms, introducing locality and sliding window strategies, achieving a good balance between accuracy and efficiency. PVT [47] further combined CNN’s pyramid feature concept, introducing deformable attention to improve multi-scale modeling capability, and has become the foundational backbone for many segmentation frameworks. Meanwhile, to address the high computational complexity of Transformer structures, Segformer [48] proposed a concise and efficient MiT encoder and achieved semantic recovery through lightweight MLP decoders, achieving excellent performance on multiple public datasets. Additionally, MaskFormer [49] modeled semantic segmentation as a unified mask prediction problem, performing pixel classification by learning the correspondence between mask embeddings and class embeddings.

The powerful long range modeling capability of Transformers has brought new solutions to crack detection. Wang et al. [50] proposed SegCrack, which for the first time used a hierarchical structured Transformer encoder as the sole encoder to extract multi-scale features, combined with a top down decoding path to progressively fuse features, and employed an online hard example mining strategy to enhance model performance. Tao et al. [51] proposed CTCrackSeg, which designed dilated residual blocks to focus on local details of the cracks and introduced a boundary aware module to learn crack boundary features, integrating a lightweight Transformer to capture global information. Li et al. [52] proposed a crack detection method based on Segformer, combining cross-entropy and Dice loss functions to enhance the detection capability of fine cracks, providing an efficient solution for crack detection on concrete and asphalt surfaces across multiple scenarios.

Although Transformer excels in global modeling and feature representation, it relies on large scale data training and tends to underfit in small sample scenarios; meanwhile, due to the lack of local inductive bias, fine grained structure modeling capability is weak, which is unfavorable for boundary or small target segmentation; furthermore, some models have high computational overhead and complex deployment, and practical applications still face challenges.

### 2.3. Crack Continuity and Topology-Aware Segmentation Methods

Beyond pixel-level accuracy, recent studies have increasingly focused on preserving the structural continuity and topological integrity of crack segmentation results, recognizing that fragmented detection can severely undermine downstream crack quantification and pavement condition assessment. A recent comprehensive review by Zhang et al. [53] systematically summarized the progress of deep learning-based crack detection from the perspectives of learning paradigms, generalizability, and dataset diversity, and explicitly identified boundary-aware refinement and topology-aware continuity preservation as two of the most critical yet unresolved challenges in this field. Shit et al. [54] proposed a topology-preserving loss function called centerlineDice, which computes the overlap between segmentation masks and their morphological skeletons, theoretically guaranteeing topology preservation up to homotopy equivalence for tubular structures including cracks. Building on this foundation, Pantoja-Rosero et al. [55] proposed TOPO-Loss, a loss function that emphasizes the correct representation of crack topology during training and introduces a continuity preservation metric for evaluation. Jing et al. [56] combined persistent homology with a U-Net architecture enhanced by Vmamba to form TopoM-CrackNet, which enforces topological constraints to maintain crack connectivity and structural integrity. Xiao et al. [57] proposed IRFusionFormer for RGB-T pavement crack segmentation, introducing a topology-based loss function to preserve crack skeleton connectivity during training.

Another line of research improves crack continuity through architectural design. Yu et al. [58] proposed a dual-path hybrid network called DSCformer, which integrates enhanced dynamic snake convolution with pyramid kernels and bidirectional learnable offset iteration, together with Segformer, to combine fine tubular topological feature extraction and global context modeling for enhancing crack continuity. Song et al. [59] proposed a lightweight continuity-aware state-space network called CONTI-CrackNet, which adopts a Multi-Directional Selective Scanning Strategy, performing bidirectional scanning along horizontal, vertical, main-diagonal, and anti-diagonal directions with gated fusion and a pixel-level global–local fusion module to strengthen global crack continuity while preserving fine details.

It is worth noting that Li et al. [60] observed that topology-aware loss approaches may lead to over-connectivity, where natural crack discontinuities are erroneously bridged during segmentation. This observation suggests that both topology-loss-based methods and architecture-based methods have their respective advantages and limitations, and that the two strategies are potentially complementary. The method proposed in this paper belongs to the architecture-based category, aiming to produce more continuous and complete crack segmentation through task-specific innovations in both the CNN and Transformer branches, while preserving the natural morphological characteristics of actual cracks.

## 3. Method

### 3.1. Overview of the Proposed Network

**Figure 1:**
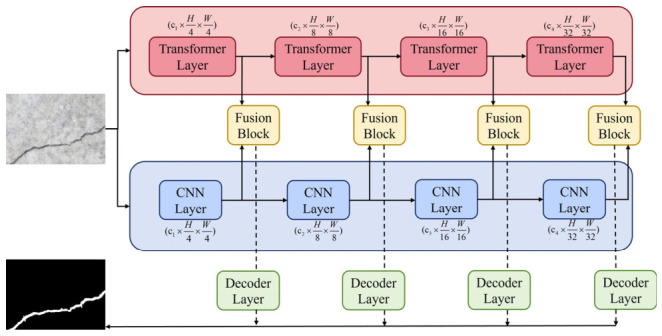
Network structure of the proposed method.

This paper proposes a crack continuity segmentation network combining Transformer and CNN. The overall network structure is shown in Figure 1, consisting of four main components: (1) a Transformer encoder; (2) a CNN encoder; (3) a hybrid attention feature fusion module; and (4) a lightweight decoder.

### 3.2. CNN Encoder

**Figure 2:**
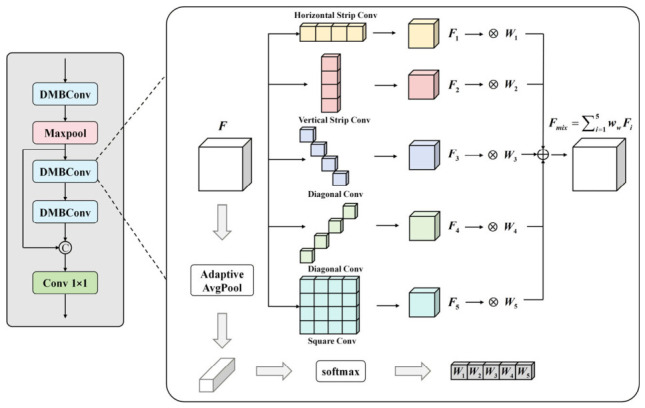
Structure of the Adaptive Directional Feature Block.

The CNN encoder consists of four layers, each of which is composed of adaptive directional feature blocks (ADFB). The directional multi-branch convolution (DMBConv) module serves as the core of each ADFB, enabling the progressive extraction of multi-directional and fine-grained semantic features of pavement cracks. The structure of a single ADFB is shown in Figure 2.

It captures information from different directions through five types of convolution kernels: $F_{1}$ employs horizontal strip shaped convolution kernels to extract horizontal crack features; $F_{2}$ uses vertical strip shaped convolution kernels focusing on vertical cracks; $F_{3}$ and $F_{4}$ respectively use irregular convolution kernels in diagonal directions to capture oblique cracks; $F_{5}$ adopts standard square convolution kernels to capture local contextual information and complement the directional branches. By extracting directional features of cracks from different geometric perspectives, the representation capability for complex crack morphologies is enhanced. Compared with kernel-deformation methods such as dynamic snake convolution, which deforms a single kernel along a continuous centerline trajectory and is best suited for coherent tubular curves, DMBConv adopts a complementary strategy by maintaining multiple fixed-shape directional kernels with adaptive weighting. This design is better aligned with the morphology of pavement cracks, which frequently exhibit branching, intersections, and abrupt orientation changes that a single deforming trajectory may struggle to capture simultaneously, and it incurs lower computational overhead since no per-position offset prediction is required.

To dynamically fuse features from these five branches, DMBConv introduces an adaptive weight learning mechanism: assuming the input feature map is $F\in R^{H\times W\times C}$, channel compressed features are obtained through a 1 × 1 convolution and global average pooling, and weight coefficients are obtained through softmax operation. A softmax operation is then used to obtain the branch weights $W=\left\{w_{1},w_{2},w_{3},w_{4},w_{5}\right\}$. The output features of the five convolutional branches are weighted by the corresponding coefficients and aggregated into the fused feature $F_{mix}=\sum_{i=1}^{5}w_{i}F_{i}$. By learning adaptive weights, the model selects the most suitable convolution shape to extract crack features.

**Figure 3:**
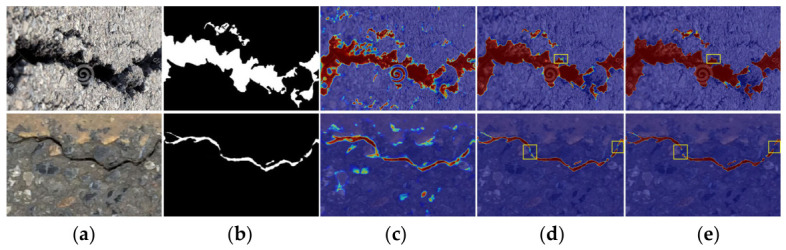
Visualization of prediction heatmaps using different convolution strategies: (**a**) Image, (**b**) GT, (**c**) convolutional layer, (**d**) DSConv, (**e**) ADFB.

Figure 3 shows the heatmap comparison of crack extraction using a standard convolutional layer, DSConv, and the proposed ADFB.

The standard convolution produces strong false responses to spiral watermarks and scattered abnormal activations in background regions in the first row, and obvious false activations on background textures in the second row, indicating poor discrimination between cracks and visually similar interference. DSConv largely suppresses these background responses by deforming the kernel along curvilinear paths, but two limitations can be observed in the highlighted yellow boxes. In the first row, the response inside the yellow box is noticeably weaker than the surrounding crack body, indicating that DSConv’s single-trajectory deformation insufficiently captures the boundary details of wide and irregular cracks. In the second row, the ground truth shows that the crack is naturally disconnected at the location marked by the yellow boxes, but DSConv erroneously bridges the gap, producing a continuous response that does not exist in the actual crack. In contrast, ADFB maintains uniformly strong responses inside the crack region in the first row and correctly preserves the natural disconnection in the second row.

### 3.3. DCNv4 Based Transformer Encoder

**Figure 4:**
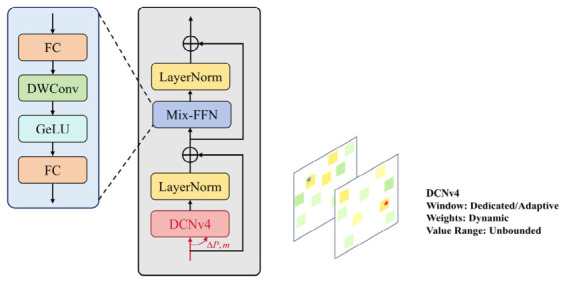
Structure of the DCNFormer. Purple star: query pixel; yellow star: response pixel.

The Transformer encoder consists of four hierarchical levels, each extracting features at different scales. First, the input image is divided into a series of 4 × 4 patches, which are then fed into the Transformer blocks. As the hierarchy deepens, neighboring patches are progressively merged and the feature map resolution is reduced, enabling multi-scale modeling. As shown in Figure 4, each Transformer Block consists of three parts: normalization layer, DCNv4 attention module, and Mix-FFN.

The traditional multi-head self-attention mechanism has the following limitations when processing crack images: (1) MHSA adopts a regular global sampling strategy, computing attention is weighted uniformly for each position, resulting in substantial computational resources being wasted on irrelevant background regions and difficulty in effectively connecting fragmented crack segments; (2) Cracks have diverse geometric morphologies, and traditional attention uses fixed position encoding, lacking the adaptive capability for geometric deformation; (3) The quadratic computational complexity of MHSA introduces substantial overhead for high-resolution images, limiting its applicability to real-time detection scenarios.

To overcome the above limitations, in each Transformer Layer an attention module constructed by DCNv4 [61] is introduced to replace the traditional multi-head attention mechanism for the feature modeling of crack images. Through learnable offsets and dynamic modulation weights, it adaptively focuses on key crack regions in images, enhancing perception capability for elongated, curved, and morphologically variable cracks. While maintaining computational efficiency, it can effectively capture long-range dependencies and complex spatial structures between crack features, enhancing continuity reconstruction capability for fragmented regions. The specific computation form of DCNv4 is as follows:

(1) $$y(p_{0})=\sum_{k=1}^{K}w_{k}\cdot x(p_{0}+p_{k}+\Delta p_{k})$$

where $p_{0}$ is the current center position, $p_{k}$ denotes the predefined grid sampling offset of the k-th point, $\Delta p_{k}$ is the learnable offset for the k-th sampling point, and $w_{k}$ is the dynamic modulation weight without softmax normalization. To further strengthen the non-linear expression of features and local spatial information fusion, Mix-FFN is connected afterwards, which introduces depth-wise separable convolution to fuse local spatial context while maintaining position encoding information. Its formula is:

(2) MixFFN(x)=FC(GeLU(DW(FC(x))))+x

where the first fully connected layer maps input features to a high dimensional space, the 3 × 3 depth-wise separable convolution performs local information aggregation in spatial dimensions, the GELU activation function introduces non-linear transformation, and the last fully connected layer maps features back to the original dimension.

**Figure 5:**
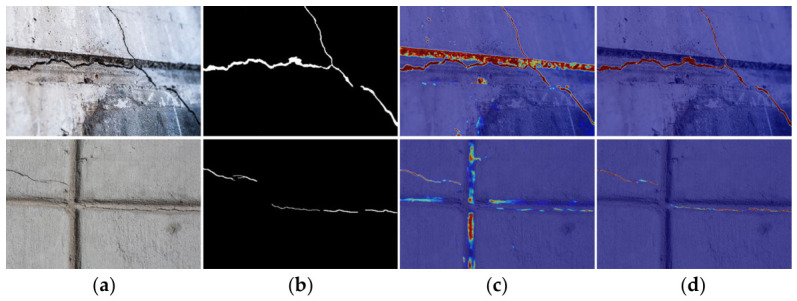
Visualization of prediction heatmaps: (**a**) Image, (**b**) GT, (**c**) MHAM, (**d**) DCNFormer.

Figure 5 shows the heatmap comparison of crack extraction using standard Transformer Block and DCNFormer Block. The standard Transformer Block misidentifies gullies in the background as cracks, while DCNFormer Block can accurately distinguish the structural textures of cracks and gullies, producing responses only at actual crack locations.

### 3.4. Fusion Block

**Figure 6:**
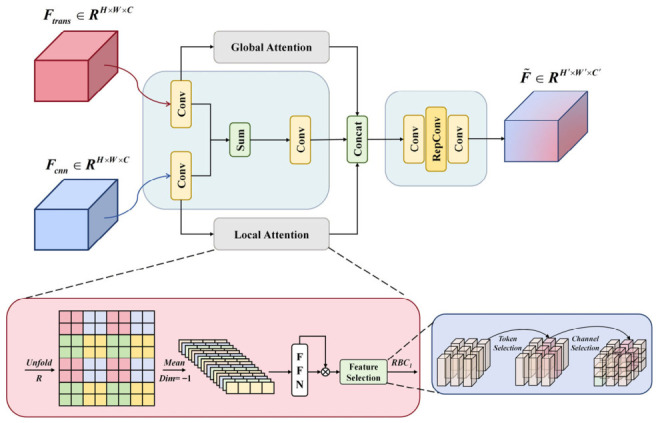
Structure of the Fusion Block.

To effectively integrate features extracted by the Transformer encoder and CNN encoder, this paper designs the fusion block. As shown in Figure 6, this method is divided into three parallel branches: local attention branch, global attention branch, and fusion branch. The fusion block receives feature $F_{cnn}\in R^{H\times W\times C}$ from the CNN branch and feature $F_{trans}\in R^{H\times W\times C}$ from the Transformer branch. In the middle path, the CNN feature and Transformer feature are first aligned in channel dimension through separate 1 × 1 convolutions, then element-wise added to produce a joint representation. This joint feature is further refined by a 3 × 3 convolutional layer to extract collaborative representations that capture the complementarity of both branches.

The global and local attention branches are used to enhance the Transformer and CNN features, respectively. The two paths share an identical computational procedure and differ only in the partition granularity parameter p. Specifically, the input feature $F\in R^{H\times W\times C}$ is first partitioned into a set of spatially contiguous and non-overlapping patch blocks of shape $\left(p\times p,H/p,W/p,C\right)$ through unfold and reshape operations. Within each patch block, the mean is computed along the channel dimension to obtain a compact descriptor of shape $\left(p\times p,H/p,W/p\right)$. After linear transformation via FFN, attention weights are obtained through softmax and applied to modulate the original features.

To further select crack-relevant information from the modulated features, a feature selection mechanism [62] is introduced to filter both spatial tokens and channels. Let d=H×W/(p×p), and represent the weighted outcome as $(t_{i}{)}_{i=1}^{d}$, where $t_{i}\in R^{C^{\prime }}$ represents the i-th output token. A learnable task embedding vector $\varepsilon \in R^{C^{\prime }}$ is introduced, and the cosine similarity $sim(t_{i},\varepsilon )$ is computed to measure the relevance of each token $t_{i}$ to the crack detection. The tokens are then reweighted as:

(3) $${\hat{t}}_{i}=P\cdot sim(t_{i},\varepsilon )\cdot t_{i}$$

where $P\in R^{C^{\prime }\times C^{\prime }}$ is a learnable channel selection matrix. The cosine similarity achieves token selection by suppressing spatially irrelevant positions, while the matrix P performs channel selection by filtering out unimportant channels. The selected features are then recovered to the original spatial dimensions through reshape and bilinear interpolation, producing $F_{\mathrm{global}}\;$ and $F_{\mathrm{local}}\;$ respectively. For global attention, p is set to 4, partitioning the feature map into $\frac{H}{4}\times \frac{W}{4}$ non-overlapping patch blocks, each covering a 4×4 spatial region. Attention is computed among the $p^{2}=16$ positions within each patch, capturing intra-patch correlations at a relatively coarse spatial granularity, which complements the Transformer’s global modeling. For local attention, p is set to 2, producing $\frac{H}{2}\times \frac{W}{2}$ patches of size 2×2, enabling finer-grained spatial attention that strengthens the CNN’s local detail extraction.

Finally, the features from the three branches are concatenated along the channel dimension and processed through a convolutional module incorporating the RepConv [63] structure. The output fused feature $\tilde{F}\in R^{H\times W\times C^{\prime }}$ achieves the complementary integration of CNN’s local perception capability and Transformer’s global modeling capability, effectively improving the detection accuracy of crack regions.

**Figure 7:**
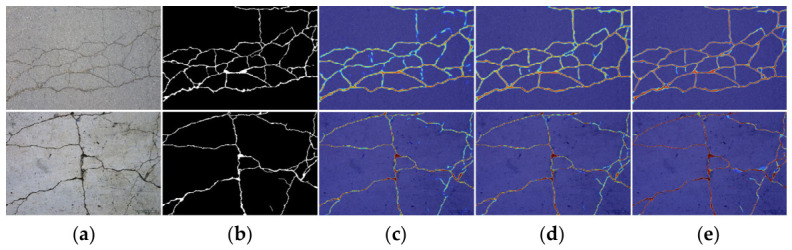
Visualization of prediction heatmaps using Transformer, CNN, and the proposed fusion module: (**a**) Image, (**b**) GT, (**c**) Transformer, (**d**) CNN, (**e**) Fusion.

Figure 7 shows the segmentation heatmap results of crack images under separate Transformer structure, separate CNN structure, and fusion structure. From the visualization results, the fusion module combines Transformer’s global modeling capability with CNN’s local detail advantages, achieving smoother, more continuous crack segmentation with clearer boundaries, demonstrating significant advantages.

### 3.5. Decoder

To achieve efficient parsing and the semantic recovery of multi-scale fused features, this paper adopts the lightweight decoder structure proposed by Segformer. First, fused features $F_{1}$, $F_{2}$, $F_{3}$, and $F_{4}\;$ from different stages are respectively processed through MLPs to unify channel dimensions, obtaining mapped features ${\tilde{F}}_{1}$, ${\tilde{F}}_{2}$, ${\tilde{F}}_{3}$, and ${\tilde{F}}_{4}$. Then the decoder applies bilinear interpolation upsampling to low resolution features ${\tilde{F}}_{2}$, ${\tilde{F}}_{3}$, and ${\tilde{F}}_{4}$ to match the spatial size of the highest resolution feature, and concatenates the four groups of features before feeding them into the fusion convolution module. This lightweight decoder has a simple and efficient structure, which aggregates multi-scale features without introducing additional computational burden. It complements the aforementioned dual branch encoder, achieving a good balance between performance and efficiency for the overall network.

### 3.6. Loss Function

Crack pixels typically occupy only a small fraction of an image, leading to a severe foreground–background class imbalance that conventional cross-entropy loss cannot handle well. To alleviate this, we adopt a joint loss combining binary cross-entropy and Dice loss, which improves recognition of crack regions while preserving pixel-level accuracy.

First, the cross-entropy function performs independent binary classification for each pixel to measure the difference between predicted probabilities and ground truth labels. Its formula is:

(4) $$L_{BCE}=-(1-t)\log(1-p)-t\log(p)$$

where t∈[0,1] represents the ground truth label and p∈[0,1] is the model’s predicted probability output. This loss term is suitable for the binary classification scenario of crack and non-crack, enabling the model to generate precise segmentation results at the pixel level. However, cross-entropy function does not consider prediction consistency between overall regions and is insensitive to small region targets.

To this end, we introduce Dice loss to better measure the overlap between predicted regions and ground truth regions, defined as:

(5) $$L_{Dice}=1-\frac{2\sum_{i=1}^{N}p_{i}t_{i}+\varepsilon }{\sum_{i=1}^{N}p_{i}+\sum_{i=1}^{N}t_{i}+\varepsilon }$$

where $p_{i}$ and $t_{i}$ are the predicted value and ground truth label for the *i*-th pixel respectively, and ε is a smoothing factor for numerical stability to prevent division by zero. The final joint loss function form is:

(6) $$L_{Total}=L_{BCE}+L_{Dice}$$

**Figure 8:**
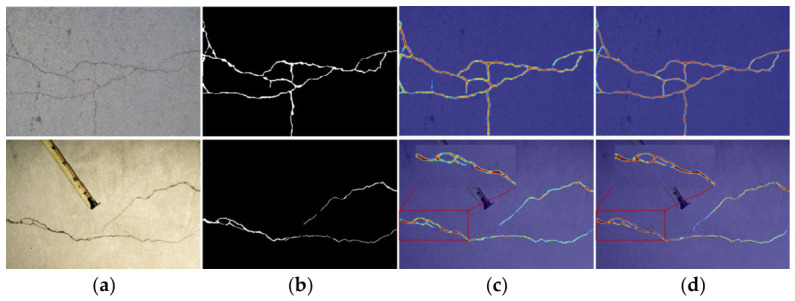
Visualization of prediction heatmaps using BCE and BCE + Dice loss functions: (**a**) Image, (**b**) GT, (**c**) BCE, (**d**) BCE + Dice.

The combined loss function effectively improves the model’s segmentation performance on crack edges and detail structures, adapting to crack recognition scenarios where complex backgrounds and tiny targets coexist. Figure 8 shows the heatmap prediction results of two representative crack images, with red boxes highlighting regions where the joint loss function effectively recovers fine crack details that BCE alone misses.

## 4. Experimental Result and Analysis

The parameter configuration for model training is shown in Table 1. Training was conducted on a server configured with CPU: Intel(R) Core (TM) i7-9700 and GPU: Nvidia GeForce RTX 4060ti. All models were implemented based on the PyTorch version 2.0.1. The optimizer used was AdamW with initial learning rate set to 1 × 10^−5^, dynamically adjusted using a cosine annealing strategy. A linear warm up strategy was introduced during the first five epochs of training to smooth learning rate changes and improve model stability. To ensure fair comparison, all baselines were retrained under our unified experimental setup.

### 4.1. Datasets

We evaluated the performance of the proposed model on four benchmark datasets: CFD [64], DeepCrack537 [65], Gaps384 [66], and Crack500 [67]. The dataset split is shown in Table 2. No data augmentation was applied to any of the four datasets to ensure direct comparability with prior studies that also train and test on original images.

(1) CFD: This dataset contains 118 urban road images captured in the field using mobile phones, mainly collected from Beijing, China. The images widely contain typical interference factors including shadows, oil stains, and water marks, providing strong support for evaluating the robustness of crack detection algorithms under complex backgrounds.

(2) DeepCrack537: The DeepCrack537 dataset proposed by Liu et al. contains 537 crack images with pixel-level annotations, with a unified image size of 544 × 384 pixels. This dataset is widely used in crack detection tasks to evaluate model generalization capability across diverse crack types and different shooting conditions. The authors provided an official split of 300/237 images.

(3) Gaps384: Gaps384 is derived from the original GAPs dataset, from which 384 images containing only cracks are selected. Each original image is cropped and zero-padded into 6 non-overlapping 640 × 544 pixel patches. The authors provided an official split of 465/5/39 images.

(4) Crack500: In this dataset, the authors captured pavement crack images of approximately 2000 × 1500 pixels using mobile phones. The authors cropped each image into 16 non-overlapping 640 × 360 regions and provided an official split of 1896/348/1124 images.

### 4.2. Performance Metrics

In this study, precision, recall, mIoU, and F-score are used as accuracy metrics for distress identification. The four metrics are calculated as shown in Equations (7)–(10), where TP, FP, and FN represent the number of true positive, false positive, and false negative pixels for the crack class, respectively. For the mIoU calculation, $TP_{i}$, $FP_{i}$, and $FN_{i}$ denote the corresponding values for the i-th class, k is the maximum class index, and k=1 for the binary crack detection task in this work.

(7) $$precision=\frac{TP}{TP+FP}$$

(8) $$recall=\frac{TP}{TP+FN}$$

(9) $$Fscore=2\times \frac{precision\times recall}{precision+recall}$$

(10) $$mIoU=\frac{1}{k+1}\sum_{i=0}^{k}\frac{TP_{i}}{TP_{i}+FP_{i}+FN_{i}}$$

Breakpoint density (BD) represents the number of endpoints per 1000 pixels of skeleton length, used to measure crack fragmentation frequency. More endpoints indicate more severe crack fragmentation. The specific calculation formula of this index is defined as follows:

(11) $$BD=\frac{Endpo\mathrm{int}s}{Skeleton\_Length}\times 1000$$

Average segment length (ASL) represents the average pixel length of each connected component, reflecting the average continuity of crack segments. Higher ASL values indicate longer average crack segments and better continuity. The specific calculation formula of this index is defined as follows:

(12) $$ASL=\frac{Skeleton\_Length}{N}$$

where N represents the number of connected components of the skeletonized crack.

Longest path ratio (LPR) represents the ratio of the longest connected component to total skeleton length, reflecting the integrity of the main crack. Ideally, when cracks are completely continuous, LPR approaches 1. The specific calculation formula of this index is defined as follows:

(13) $$LPR=\frac{Longest\_Component\_Length}{Skeleton\_Length}$$

Fragmentation index (FI) is used to measure the fragmentation degree of crack segmentation results, with value range [0, 1]. When there is only one connected component, FI = 0, indicating completely continuous cracks; more connected components result in FI closer to 1, indicating more severe fragmentation. The specific calculation formula of this index is defined as follows:

(14) $$FI=1-\frac{1}{NCC}$$

where NCC represents the number of connected components. Continuity index (CI) is a comprehensive metric that integrates information from LPR, FI, and normalized breakpoint density, comprehensively evaluating crack continuity from multiple dimensions. The specific calculation formula of this index is defined as follows:

(15) $$CI=LPR\times (1-FI)\times (1-BD_{norm})$$

where ${BD}_{norm}=min(BD/100,1)$ is the normalized breakpoint density. CI ranges from 0 to 1, where higher values indicate better continuity.

### 4.3. Comparison Experiment

Experiments compared the performance of UNet [29], HRNet [68], Segformer [48], Segmenter [69], Deepcrack [70], DTrCNet [71], DCSNet [72], Crackmer [73], and the proposed method on four public crack datasets. As shown in Table 3, the proposed method achieves a favorable balance between model complexity and segmentation accuracy, with 13.78 M parameters and 5.24 G GFLOPs. Although its inference speed is not the highest among all compared models, it reaches 60.1 FPS, which satisfies the real-time requirements of practical crack detection.

Meanwhile, Table 4 demonstrates that the proposed method achieves the best performance among all compared methods in F-score and mIoU across all four datasets. On the CFD dataset, our method achieves an F-score and mIoU of 82.75% and 84.63% respectively, representing improvements of 4.74% and 3.49% over the second-best method, Crackmer. On the Gaps384 dataset, our method achieves an F-score of 77.36% and mIoU of 81.03%, with improvements of 9.09% and 5.62% over Crackmer, showing the most significant performance gains. On the DeepCrack537 dataset, our method achieves an F-score and mIoU of 88.71% and 88.80% respectively, improving by 1.52% and 1.37% over Crackmer. On the Crack500 dataset, our method achieves an F-score of 83.03% and an mIoU of 84.21%, improving the F-score by 0.46% over the second-best Deepcrack and the mIoU by 1.86% over the second-best Crackmer.

**Figure 9:**
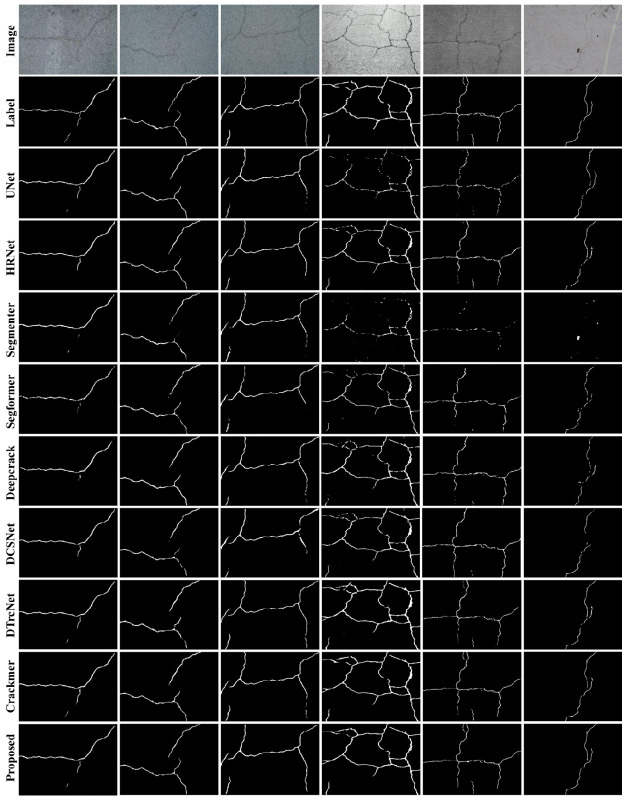
Illustration of segmentation results on CFD and DeepCrack537 datasets.

**Figure 10:**
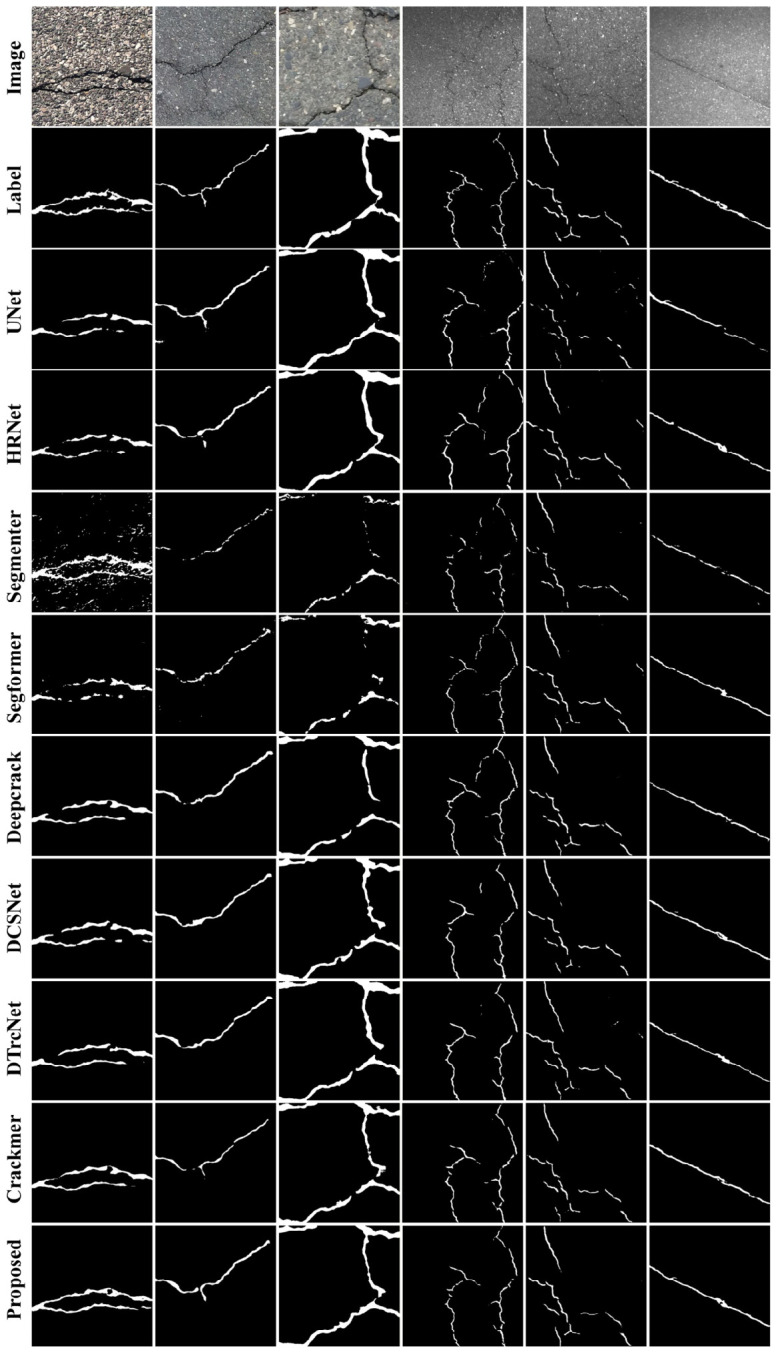
Illustration of segmentation results on Crack500 and Gaps384 datasets.

The visualization results in Figure 9 and Figure 10 further reveal the segmentation characteristics of each method. UNet and HRNet can capture the main structure of cracks, but show obvious discontinuities when segmenting cracks, especially missed detections at crack branch intersections.

Segmenter has the worst overall performance, with large missed detections and severely discontinuous segmentation results. Segformer also has unsatisfactory crack continuity, with serious missed detections of low-contrast fine cracks, and its overall performance is even worse than UNet and HRNet. Deepcrack enhances detection performance through multi-scale feature fusion but lacks precise boundary localization in complex networked crack scenarios. DCSNet, DTrCNet, and Crackmer, as specialized crack detection networks, show better overall performance but still exhibit local discontinuities or boundary adhesion issues when dealing with densely branched cracks. In contrast, the proposed method maintains better crack continuity across various scenarios and achieves more complete recognition of branching structures in network-like cracks.

### 4.4. Model Continuity Comparison Experiment

**Figure 11:**
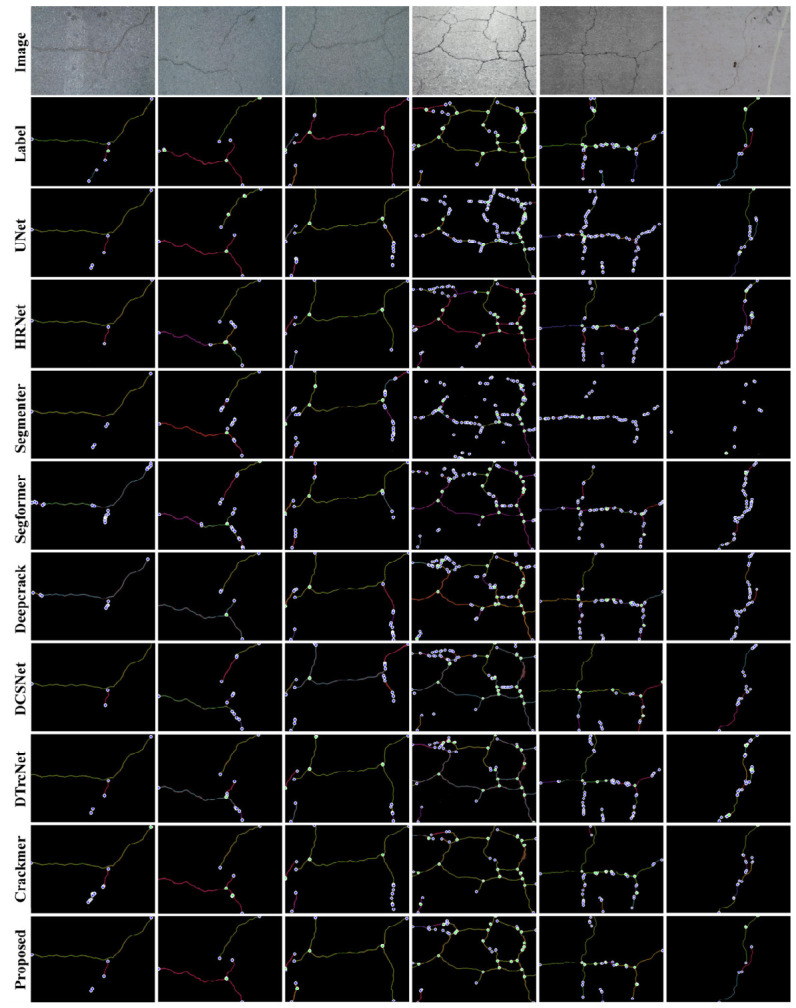
Illustration of continuity segmentation results on CFD and DeepCrack537 datasets.

**Figure 12:**
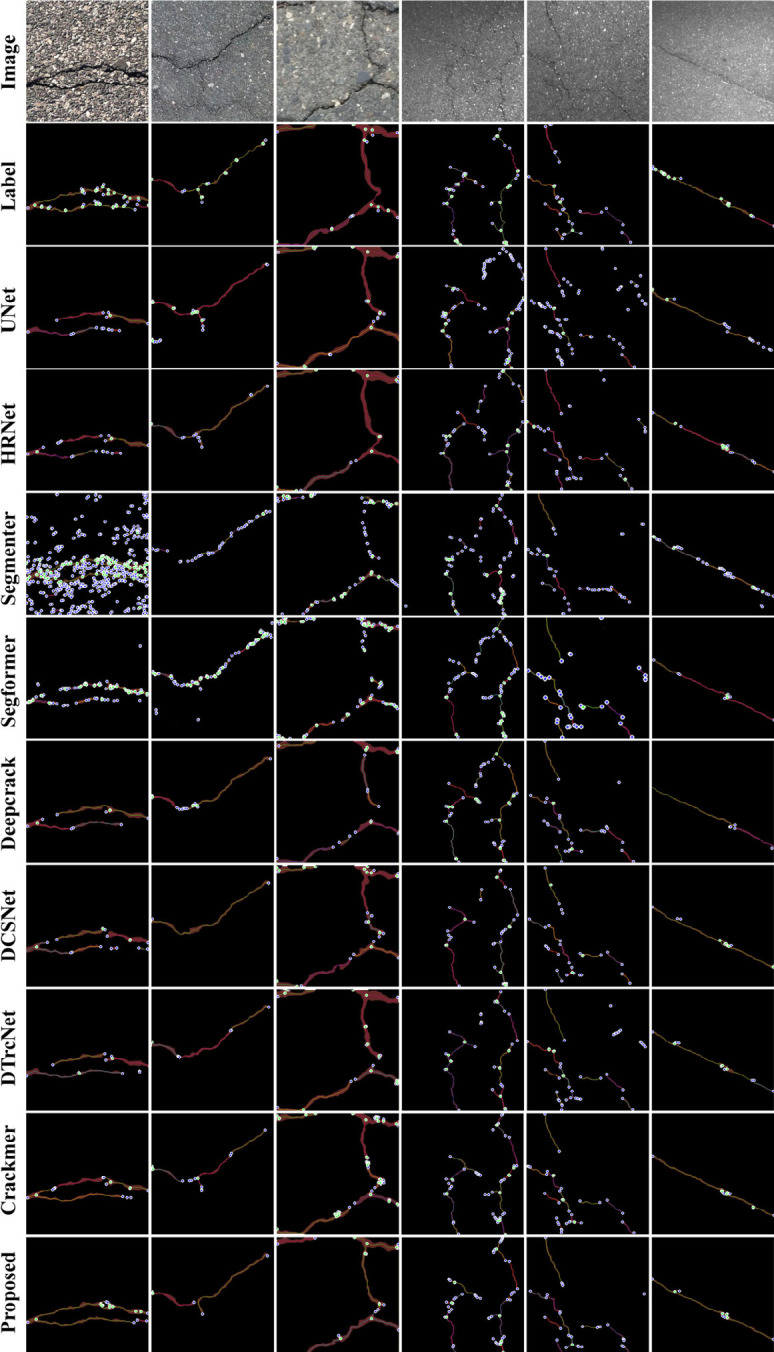
Illustration of continuity segmentation results on Crack500 and Gaps384 datasets.

Figure 11 and Figure 12 show the continuity comparison results of different crack segmentation methods. Different colored line segments represent different connected components; more colors indicate more fragmented crack segmentation. Blue circles mark endpoint positions, representing crack break points; green circles mark junction points, representing intersections of multiple cracks. Before computing the continuity metrics, the predictions of all methods were first binarized using a fixed threshold of 0.5 obtained via argmax over the two-class softmax output, and the resulting binary masks were skeletonized using the Zhang–Suen thinning algorithm, with no morphological post-processing applied to any method’s outputs. Since all methods are processed under identical conditions, these factors affect all methods equally and do not introduce bias in the relative comparison.

To quantitatively evaluate the crack continuity performance, the proposed continuity metrics were computed on every test image across all four datasets. Table 5 reports the average results over all test images from the four datasets. Segmenter and Segformer generate a large number of independent short line segments and dense endpoint markers, with BD values of 70.80 and 51.01, FI values of 0.63 and 0.61, and CI values of only 0.04 and 0.07, respectively, exhibiting severe over-segmentation problems. Methods such as UNet, HRNet, and Deepcrack show moderate fragmentation in the visualization results, with BD values ranging from 22 to 45 and CI values ranging from 0.12 to 0.18. Although they outperform Segmenter, obvious discontinuities still exist. DCSNet, DTrCNet, and Crackmer maintain relatively good continuity in the figures, but fracture phenomena can still be observed near narrow and elongated cracks and intersection points. The method proposed in this paper produces the fewest crack fractures and obtains the most complete main crack path. With a BD of only 15.46, it achieves the lowest value among all methods; the ASL reaches 314.56, which is significantly higher than other methods; the LPR is 0.64, the FI is 0.36, and the CI reaches 0.35, all achieving the best values among all compared methods.

### 4.5. Branch Ablation Experiment

To further examine the contribution of each architectural component, this section reports ablation experiments from the following three perspectives: (1) the comparison between the complete dual-path framework and its single-branch variants, which verifies the complementarity between the CNN and Transformer paths; (2) the comparison among different Transformer encoders, which evaluates the effectiveness of the proposed DCNv4-based Transformer branch; and (3) the comparison among several representative CNN backbones, which validates the advantage of the proposed directional CNN encoder in modeling elongated crack structures.

#### 4.5.1. Ablation on the Dual-Path Architecture

To validate the effectiveness of the proposed dual-path encoding structure, we conducted ablation experiments on four public datasets, with results shown in Table 6. The dual-path structure outperformed single structures using only CNN or Transformer on all four datasets.

On the CFD dataset, the proposed model achieved an F-score of 82.75% and an mIoU of 84.63%, the highest among the three structures. Although the Transformer-only branch obtained a recall of 87.32%, which was 1.87% higher than that of the proposed model, its precision was only 77.03%. The CNN-only branch also performed worse than the proposed model, with an F-score of 79.72%.

On the Gaps384 dataset, the proposed model achieved an F-score of 77.36% and an mIoU of 81.03%, while the CNN-only branch achieved an F-score of only 66.53%, which was 10.61% lower than that of the proposed method.

On the DeepCrack537 dataset, the proposed method achieved an F-score of 88.71% and an mIoU of 88.80%. Although the Transformer-only branch obtained a higher recall of 91.67%, its precision was only 85.52%, and its mIoU decreased to 86.94%.

On the Crack500 dataset, the proposed model achieved an F-score of 83.03% and an mIoU of 84.21%. The Transformer-only variant obtained an F-score of 77.80% and an mIoU of 80.23%, while the CNN-only variant achieved an F-score of 79.83% and an mIoU of 81.77%. Although the CNN-only variant showed slightly higher precision than the proposed model, its recall was substantially lower, indicating that the dual-path structure improves crack completeness while maintaining competitive precision.

In summary, the proposed dual-path structure combines the local texture sensitivity of CNNs with the global semantic modeling capability of Transformers, thereby improving crack detection accuracy, completeness, and continuity. It effectively alleviates the fragmented segmentation problem that often occurs in single-branch architectures.

#### 4.5.2. Comparison of Different Transformer Encoders

To evaluate the feature modeling capability of different Transformer encoders, ViT, PVT, Swin Transformer, and the proposed DCNv4-based encoder were compared. The experimental results are shown in Table 7.

On CFD and Gaps384 datasets, the proposed method outperformed other models on all four metrics. On the CFD dataset, precision reached 80.21%, recall was 85.45%, F-score reached 82.75%, and mIoU was 84.63%; in comparison, the closest performing Swin Transformer had mIoU of 82.27%, 2.36% lower than the proposed method. On Gaps384, the proposed model’s mIoU reached 81.03%, the highest among all models. On the DeepCrack537 dataset, although overall performance of all models was generally high, the proposed method still achieved the highest recall and mIoU among all compared methods. On the Crack500 dataset, the proposed method also achieved the best performance across all four metrics.

**Figure 13:**
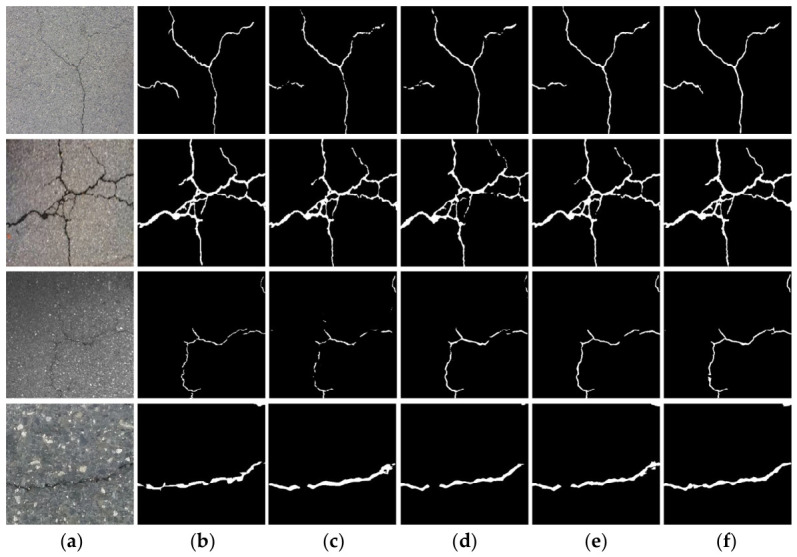
Different Transformer encoder recognition results: (**a**) Image, (**b**) GT, (**c**) VIT, (**d**) PVT, (**e**) Swin Transformer, (**f**) Proposed.

In summary, the Transformer encoder designed in this paper has obvious advantages in comprehensive performance, demonstrating good adaptability and generalization capability in crack segmentation tasks. Recognition results of different Transformer encoders are shown in Figure 13.

#### 4.5.3. Comparison of Different CNN Encoders

To evaluate the impact of different CNN encoder structures on crack segmentation performance, we selected three representative classic architectures, MobileNetv3 [74], VGG [11], and ResNet [75], for comparison with the proposed encoder. Experimental results are shown in Table 8.

On the CFD dataset, the proposed method achieved the best performance among all compared methods on all metrics, with an F-score of 82.75% and an mIoU of 84.63%. In comparison, ResNet performed second best, with an F-score of 79.35% and an mIoU of 82.28%.

On the Gaps384 dataset, the proposed method’s mIoU was 81.03%, 3.62%, and 3.78% higher than ResNet and VGG respectively. MobileNetv3 showed relatively lower precision and recall, possibly due to its limited representation capacity under the lightweight architecture. On the DeepCrack537 dataset, the proposed method achieved an F-score of 88.71% and an mIoU of 88.80%. Although VGG’s recall was 88.68%, its F-score and mIoU were 1.17% and 1.03% lower than the proposed method respectively.

On the Crack500 dataset, the proposed method achieved an F-score of 83.03% and an mIoU of 84.21%. ResNet performed second best, with an F-score of 81.59% and an mIoU of 83.06%. VGG followed with an F-score of 80.80% and an mIoU of 82.46%, which were 2.23% and 1.75% lower than those of the proposed method, respectively. MobileNetv3 showed the lowest performance, with an F-score of 80.18% and an mIoU of 82.00%, mainly due to its limited representation capacity under the lightweight architecture.

**Figure 14:**
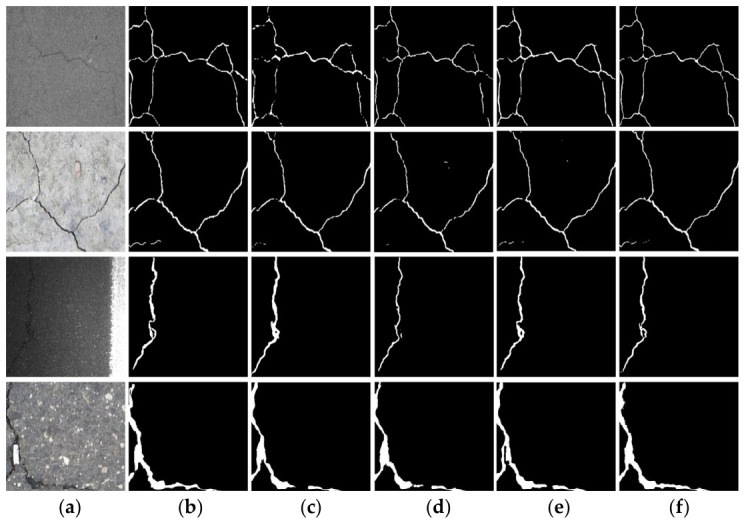
Different CNN encoder recognition results: (**a**) Image, (**b**) GT, (**c**) MobileNetv3, (**d**) VGG, (**e**) ResNet, (**f**) Proposed.

In summary, the designed CNN encoder not only significantly improves crack detection accuracy, but also performs excellently in preserving tiny cracks and restoring complex edge details, ensuring the completeness and stability of segmentation results. Recognition results of different CNN encoders are shown in Figure 14.

## 5. Discussion

The continuity of crack detection not only affects visual appearance but also directly relates to the accuracy of pavement condition evaluation. Pavement condition index (PCI) is a widely adopted evaluation metric in the road maintenance field. According to China’s “Highway Performance Assessment Standard (JTG 5210-2018)” [76], the quantitative evaluation of PCI mainly relies on crack length and area measurements. The evaluation model is mainly based on the functional relationship between PCI and DR:

(16) $$PCI=100-a_{0}DR^{a_{1}}$$

(17) $$DR=100\times \frac{\sum_{i=1}^{i_{0}}w_{i}A_{i}}{A}$$

where DR is the comprehensive pavement damage rate, which is the percentage of the sum of equivalent areas of various distresses to the surveyed pavement area; i is the distress type; $i_{0}$ is the total number of distress types; $A_{i}$ is the damaged area of the i-th type of pavement distress; A is the surveyed pavement area (product of survey length and pavement width); $w_{i}$ is the weight of the i-th type of pavement distress. For asphalt pavement, $a_{0}$ is 15.00 and for cement pavement is 10.66; asphalt pavement uses $a_{1}$ of 0.412 and cement pavement uses 0.461.

**Figure 15:**
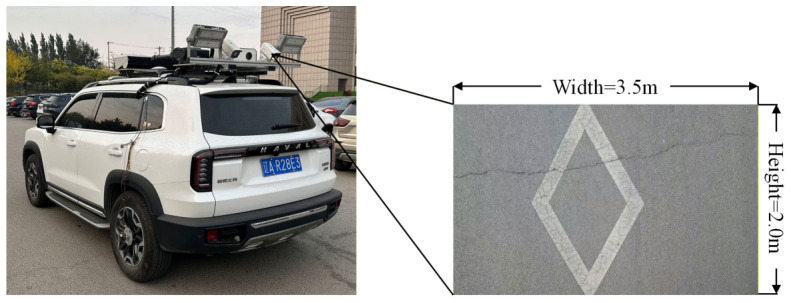
Data acquisition equipment.

To verify the practical engineering value of the proposed method in crack continuity detection, field experiments were conducted on three 2-km road sections in Fuxin City, Liaoning Province. Pavement images were captured by industrial cameras mounted on vehicle rooftops, as shown in Figure 15. The cameras were calibrated using the checkerboard calibration method to obtain intrinsic parameters and distortion coefficients, and the collected images were ortho-rectified through a homography matrix. The corrected image resolution was 3200 × 1800 pixels, corresponding to actual pavement dimensions of 3.5 m × 2 m, yielding a pixel to actual distance conversion coefficient of 1.1 mm/pixel.

**Figure 16:**
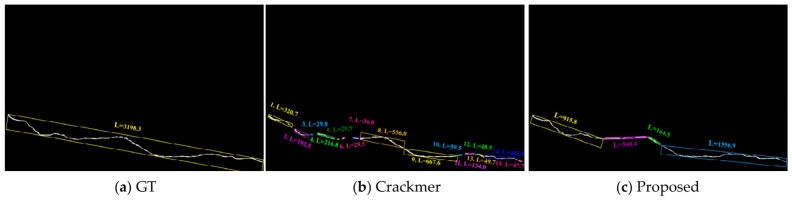
Comparison of the minimum external rectangle of crack detection results by Crackmer and Proposed.

In Figure 16, the ground truth crack total length was 3198.3 px, the proposed method calculated 3185.6 px, with an error of only 0.4% from the ground truth; while the Crackmer method, due to breaking the crack into multiple segments and calculating bounding rectangles separately, obtained a total length of only 2875.2 px, underestimating by 11.7% compared to the ground truth. In engineering practice, the length of each detected crack segment is approximated by the longer side of its minimum bounding rectangle, and the overall crack length is obtained by summing the lengths of all segments. When a continuous crack is broken into disconnected pieces, the gaps between them are not included; therefore, higher BD, lower ASL, and lower LPR all lead to greater cumulative length underestimation.

To verify that this length-based causality is not an artifact of a particular threshold choice, we conducted a sensitivity experiment by varying the binarization threshold from 0.3 to 0.7 and computing both the continuity metrics and the resulting crack length error for the proposed method and Crackmer on the same set of field validation images. Table 9 reports the full set of quantitative results across all thresholds. The results confirm that the proposed method consistently outperforms Crackmer under every threshold, with stable continuity metrics and crack length error remaining below 2.0% in all cases.

Based on the above crack length measurement results, the pavement condition index (PCI) was further calculated to verify how length measurement errors propagate into pavement condition assessment. Since $A_{i}$ is directly proportional to the measured crack length as described above, any length underestimation caused by fragmentation reduces $A_{i}$ proportionally, lowers the weighted sum ${w_{i}A}_{i}$, decreases DR, and inflates the final PCI score, leading to an overly optimistic assessment of pavement condition. To quantitatively verify this effect, three experts in the distress inspection field used manual visual interpretation to accurately annotate pavement distresses in evaluation unit images and calculated corresponding PCI scores as ground truth.

As shown in Table 10, across the three test road sections, the average PCI difference for the proposed method is only 0.81, while the average difference for the Crackmer method reaches 1.90. The Crackmer method consistently yields higher PCI values than the ground truth, which is fully consistent with the expected effect of length underestimation. This confirms that the proposed continuity metrics have a direct and quantifiable impact on PCI evaluation: poorer continuity reflected by higher BD, lower ASL, and lower LPR indicates more severe fragmentation, which shortens the measured crack length and inflates PCI, whereas better continuity yields more reliable PCI scores.

It should be noted that the present PCI-level validation was conducted on three 2-km asphalt sections within a single urban area, under relatively consistent weather and illumination conditions. Although the observed average PCI deviation of 0.81 is encouraging, the sample size remains limited and does not cover cement pavements, severe weather (rain, snow, ice), night-time illumination, or heavily aged sections with composite distresses such as potholes and patches. Larger-scale, multi-region, and multi-seasonal validation is therefore required before the proposed method can be considered fully qualified for production-grade deployment. Extending the field study to such conditions constitutes an important part of our future work.

## 6. Conclusions

This paper proposes a dual-path crack continuity segmentation network that integrates CNN and Transformer. Through the dual-path architecture, the proposed network simultaneously captures local detailed features and global contextual information. In the CNN branch, a dynamic multi-branch convolution module is designed to enhance directional perception for elongated cracks; in the Transformer branch, the DCNv4 module is employed to adaptively focus on critical crack regions and model long-range spatial dependencies. The fusion block integrates dual-path features through three parallel pathways of global attention, local attention, and feature selection, effectively enhancing the structural continuity of crack regions. Additionally, a joint loss function combining binary cross-entropy and Dice loss is adopted to alleviate the severe class imbalance between positive and negative samples, further improving the fine-grained recognition capability at crack edges. Quantitative evaluation and qualitative analysis are performed on four public datasets including CFD, Gaps384, DeepCrack537, and Crack500. Results show that the proposed model outperforms existing mainstream methods in terms of F-score and mIoU and generates more continuous and complete segmentation outputs. Ablation studies further validate that the dual-path structure and fusion mechanism play a critical role in improving the segmentation accuracy of continuous cracks.

However, several limitations remain to be addressed in future work. Although our model achieves approximately 60 FPS with 5.24 GFLOPs, its dual-path parallel CNN–Transformer design may hinder deployment on extremely resource-constrained edge devices. More importantly, this work follows most existing crack segmentation methods by treating pixel-level annotations as ground truth, without explicitly considering label noise and annotation uncertainty. In practice, crack annotations are often subjective, especially around boundaries, tips, thin branches, and faint-texture regions, which are also crucial for continuity-aware segmentation. Although the BCE + Dice loss can alleviate the influence of minor boundary deviations to some extent, it does not provide a formal noise-aware learning mechanism. Future work will therefore explore uncertainty-aware selective training and prediction, loss correction, and consistency-based regularization to improve segmentation reliability in ambiguous regions and provide more trustworthy support for automated pavement condition assessment.

## Figures and Tables

**Table 1:** The parameter settings.

Parameter	Parameter Settings
Epoch	300
Batchsize	4
Momentum	0.9
Initial learning rate	1 × 10^−5^
Optimizer	AdamW
Weight decay	1 × 10^−4^
Seed	11
Learning rate decay type	Cos

**Table 2:** Pavement crack segmentation dataset split.

Datasets	Train	Val	Test
CFD	83	-	35
DeepCrack537	300	-	237
Gaps384	465	5	39
Crack500	1896	348	1124

**Table 3:** Comparison of model complexity and inference efficiency.

Model	Parameters/M	GFLOPs	FPS
UNet	24.89 M	56.46 G	63.0
HRNet	29.54 M	11.37 G	44.5
Segmenter	25.34 M	7.17 G	184.4
Segformer	27.35 M	28.36 G	86.9
Deepcrack	30.91 M	137.46 G	23.5
DCSNet	15.19 M	8.60 G	192.0
DTrCNet	41.83 M	40.84 G	63.7
Crackmer	9.63 M	2.38 G	74.3
Proposed	13.78 M	5.24 G	60.1

**Table 4:** Comparison of different methods on the four datasets.

Model	CFD				DeepCrack537				Gaps384				Crack500			
	P	R	F	mIoU	P	R	F	mIoU	P	R	F	mIoU	P	R	F	mIoU
UNet	74.50	67.69	70.93	74.18	86.36	83.50	84.91	85.54	53.83	75.08	62.70	72.14	86.39	75.01	80.30	82.15
HRNet	73.76	63.51	68.25	75.07	81.03	87.67	84.22	84.87	66.01	65.51	65.76	73.96	79.83	74.62	77.14	79.73
Segmenter	71.53	53.88	61.46	71.23	78.56	85.71	81.98	82.98	57.17	59.71	58.41	69.97	78.00	75.20	76.57	79.54
Segformer	71.49	60.17	65.34	73.36	78.45	88.75	83.28	84.05	60.80	60.54	60.67	70.67	80.85	75.01	77.82	80.23
DeepCrack	74.32	61.55	67.33	74.53	84.71	84.05	84.38	85.08	59.20	62.05	60.59	71.10	82.47	82.66	82.56	81.83
DCSNet	78.47	76.64	77.54	81.04	89.86	72.46	80.23	81.80	63.71	67.04	65.33	73.70	83.07	81.50	82.28	81.62
DTrCNet	77.66	76.56	77.11	80.67	86.83	85.71	86.27	86.58	68.30	68.78	68.54	75.49	80.52	80.31	80.41	82.15
Crackmer	76.56	79.52	78.01	81.14	86.04	88.37	87.19	87.43	67.87	68.67	68.27	75.41	84.84	76.74	80.59	82.35
Proposed	80.21	85.45	82.75	84.63	87.42	90.03	88.71	88.80	75.94	78.84	77.36	81.03	83.46	82.60	83.03	84.21

**Table 5:** Results of the continuity comparison experiment.

Model	BD	ASL	LPR	FI	CI
UNet	44.95	131.57	0.47	0.52	0.12
HRNet	22.11	160.71	0.48	0.52	0.18
Segmenter	70.80	47.04	0.37	0.63	0.04
Segformer	51.01	82.01	0.39	0.61	0.07
Deepcrack	26.20	128.00	0.49	0.52	0.17
DCSNet	20.36	196.11	0.56	0.44	0.25
DTrCNet	21.34	173.52	0.53	0.47	0.22
Crackmer	19.20	202.85	0.57	0.44	0.26
Proposed	15.46	314.56	0.64	0.36	0.35

**Table 6:** Comparison of the effectiveness of CNN encoder and Transformer encoder, where a denotes the Transformer-only branch, and b denotes the CNN-only branch.

Model	CFD				DeepCrack537				Gaps384				Crack500			
	P	R	F	mIoU	P	R	F	mIoU	P	R	F	mIoU	P	R	F	mIoU
a	77.03	87.32	81.85	81.92	85.52	91.67	88.49	86.94	67.74	70.78	68.86	75.76	81.36	74.54	77.80	80.23
b	76.93	82.71	79.72	82.20	86.59	89.11	87.83	88.01	66.60	66.46	66.53	77.84	84.04	76.03	79.83	81.77
Proposed	80.21	85.45	82.75	84.63	87.42	90.03	88.71	88.80	75.94	78.84	77.36	81.03	83.46	82.60	83.03	84.21

**Table 7:** Comparison of the effectiveness of different Transformer encoders, where ST denotes Swin Transformer.

Model	CFD				DeepCrack537				Gaps384				Crack500			
	P	R	F	mIoU	P	R	F	mIoU	P	R	F	mIoU	P	R	F	mIoU
ViT	76.52	79.38	77.92	81.12	85.95	87.22	86.58	86.94	72.18	73.92	73.04	78.34	82.69	77.51	80.02	81.89
PVT	76.58	81.32	78.88	81.94	86.16	88.11	87.12	87.40	71.37	75.70	73.47	78.61	82.48	78.87	80.63	82.34
ST	77.08	81.70	79.33	82.27	85.97	87.47	86.71	87.05	71.72	77.54	74.52	79.28	81.82	80.81	81.31	82.85
Proposed	80.21	85.45	82.75	84.63	87.42	90.03	88.71	88.80	75.94	78.84	77.36	81.03	83.46	82.60	83.03	84.21

**Table 8:** Comparison of the effectiveness of different CNN encoders, where M3 denotes MobileNetv3.

Model	CFD			DeepCrack537					Gaps384				Crack500			
	P	R	F	mIoU	P	R	F	mIoU	P	R	F	mIoU	P	R	F	mIoU
M3	75.40	77.69	76.52	80.31	85.62	87.56	86.58	86.93	68.67	70.36	69.50	76.15	82.78	77.74	80.18	82.00
VGG	77.38	80.46	78.89	81.96	86.44	88.68	87.54	87.77	69.92	72.74	71.30	77.25	82.22	79.42	80.80	82.46
ResNet	77.21	81.61	79.35	82.28	85.97	88.68	87.30	87.55	69.66	73.59	71.57	77.41	82.29	80.89	81.59	83.06
Proposed	80.21	85.45	82.75	84.63	87.42	90.03	88.71	88.80	75.94	78.84	77.36	81.03	83.46	82.60	83.03	84.21

**Table 9:** Sensitivity of continuity metrics and crack length error to binarization threshold.

Threshold	Method	BD	ASL	CI	Crack Length Error (%)
0.3	Crackmer	20.83	186.72	0.23	8.9
0.3	Proposed	17.82	278.91	0.30	1.5
0.4	Crackmer	19.72	196.41	0.25	10.2
0.4	Proposed	16.53	296.73	0.33	0.8
0.5	Crackmer	19.20	202.85	0.26	11.7
0.5	Proposed	15.46	314.56	0.35	0.4
0.6	Crackmer	21.35	182.47	0.22	13.5
0.6	Proposed	16.91	289.15	0.32	1.1
0.7	Crackmer	24.18	158.92	0.18	16.1
0.7	Proposed	18.74	261.38	0.28	2.0

**Table 10:** PCI values calculated by different methods.

PCI			Difference	
GT	Crackmer	Proposed	Crackmer	Proposed
92.07	93.63	92.59	1.56	0.52
86.46	88.51	87.59	2.05	1.13
90.21	92.30	90.98	2.09	0.77

## Data Availability

The datasets generated in this study are available from the corresponding author upon reasonable request.
